# Inflammatory cytokines and change of Th1/Th2 balance as prognostic indicators for hepatocellular carcinoma in patients treated with transarterial chemoembolization

**DOI:** 10.1038/s41598-019-40078-8

**Published:** 2019-03-01

**Authors:** Hae Lim Lee, Jeong Won Jang, Sung Won Lee, Sun Hong Yoo, Jung Hyun Kwon, Soon Woo Nam, Si Hyun Bae, Jong Young Choi, Nam Ik Han, Seung Kew Yoon

**Affiliations:** 1 Department of Internal Medicine, Bucheon St. Mary’s hospital, College of Medicine, The Catholic University of Korea, Seoul, Republic of Korea; 2 Department of Internal Medicine, Seoul St. Mary’s hospital, College of Medicine, The Catholic University of Korea, Seoul, Republic of Korea; 3 Department of Internal Medicine, Incheon St. Mary’s Hospital, College of Medicine, The Catholic University of Korea, Seoul, Republic of Korea; 4 Department of Internal Medicine, St. Paul’s hospital, College of Medicine, The Catholic University of Korea, Seoul, Republic of Korea

## Abstract

Tumor-associated immune response plays a critical role in cancer pathogenesis. This study evaluated clinical implications of T cell cytokines and regulatory T cells (Tregs) in HCC patients treated with TACE. Whole blood was obtained for analysis of T cell cytokines (IL-1β, IL-2, IL-4, IL-5, IL-6, IL-9, IL-10, IL-12p70, IL-13, IL-17A, IL-22, IFN-γ, and TNF-α) and Tregs from 142 HCC patients. Patients with CTP class A had a significantly lower proportion of detectable IL-4 or IL-6, but a higher proportion of detectable IL-22 than patients with CTP class B/C. IL-6 level was well correlated with tumor stage and undetectable IL-17A was associated with extrahepatic metastasis. The overall survival rate was significantly higher in patients who had undetectable IL-6 or detectable IL-22 than patients who did not. IL-6 among cytokines remained independently predictive factor for survival. Increased IFN-γ/IL-10 ratio and no increase in IL-6 level following TACE were associated with prolonged survival, and baseline Tregs could affect Th1/Th2 balance. T cell cytokines are associated with a variety of clinical aspects of HCC, and IL-6 is the most significant predictor of survival. A shift toward increased Th1 response and no increase in IL-6 level exert favorable immunologic effects on HCC prognosis.

## Introduction

Hepatocellular carcinoma (HCC), one of the most common cancers worldwide, usually develops from chronically inflamed or cirrhotic liver caused by hepatitis viral infection or alcohol-induced liver damage^1^. Complex interactions between tumor and host immune-inflammatory responses, enacted by both innate and adaptive immune cells and cytokines, affect cancer progression and prognosis^2^.

CD4+ helper T (Th) cells mainly participate in tumor immunology and are functionally subdivided into different subsets, Th1, Th2, and Th17 cells, according to secretory cytokines and immunologic roles^3^. Th1 cytokines (IL-1β, IL-2, IL-12, TNF-α, and IFN-γ) are associated with good prognosis in patients with HCC, whereas Th2 cytokines (IL-4, IL-5, and IL-10) are related to tumor growth or metastasis^4,5^. Although there is uncertainty about the role of Th17 cells secreting IL-17, IL-21, and IL-22, there is evidence of Th17 cells contributing to HCC progression^6,7^. Regulatory T cells (Tregs) are typically immune-suppressive, and the prevalence of Tregs is strongly correlated with HCC progression^8^.

However, study results about the pathophysiologic roles of cytokines and their clinical relevance in several cancers vary depending on disease status or study subjects because of pleiotropic cytokine features^9^. There are also interactions within immunologic components; for example, IL-6 can promote Th17 cell differentiation, and Tregs can suppress the anti-tumor response of effector T cells^10,11^. Therefore, simultaneous and sequential assessment of tumor immune cytokines is necessary to better understand the interplay between tumor and host immune-inflammatory responses that determine patient outcomes.

In this study, we comprehensively investigated the potential roles of multiple CD4+ T cell cytokines (Th1, Th2, and Th17 cell cytokines) and Tregs as factors representing tumor status and predicting prognosis in patients with HCC treated by transarterial chemoembolization (TACE). We also evaluated changes in the balance of CD4+ T cell subsets following TACE.

## Results

### Baseline characteristics

The clinical characteristics and serum levels of multiple T cell cytokines and Tregs (%) of 142 patients with HCC treated by TACE are shown in Table 1. Mean age was 60.4 ± 11.0 years, and HBV infection (71.1%) was the most prevalent cause of HCC. At enrollment, mean tumor size was 8.0 ± 6.5 cm, and nearly half of patients (49.3%) had a solitary tumor. Thirty-six (25.7%) patients had portal vein thrombosis (PVT), and 22 (15.7%) had extrahepatic metastasis. Eleven (7.7%) of 142 patients underwent TACE combined with radiotherapy while the others were treated only with TACE.

**Table 1:** Baseline characteristics and cytokine levels of HCC patients treated with TACE.

Characteristics	Total patients (n = 142)	
Gender (male : female)	114 : 28	
Age (years)	60.4 ± 11.0	
Etiologies (HBV/HCV/Alcohol/Others, %)	101/13/15/14 (71.1/9.2/10.6/9.9)^†^	
Platelet counts (×10^3^/mm^3^)	156.1 ± 95.3	
Total bilirubin (mg/dL)	1.3 ± 1.1	
Albumin (g/dL)	3.6 ± 0.6	
Prothrombin time (INR)	1.2 ± 0.2	
Child-Pugh class (A/B/C, %)	102/33/3 (73.9/23.9/2.2)	
AFP (ng/mL), median (range)	57.6 (1.3‒3.4 × 10^5^)	
PIVKA-II (mAU/mL), median (range)	185 (10‒7.5 × 10^4^)	
Tumor size (cm)	8.0 ± 6.5	
Tumor number, solitary (%)	69/140 (49.3)	
PVT (%)	36/140 (25.7)	
Extrahepatic metastasis (%)	22/140 (15.7)	
mUICC stage (I/II/III/IV, %)	18/49/29/44 (12.9/35.0/20.7/31.4)	
**Cytokines, lower limit**	**Detectable cases, n (%)**	**Median (range) value in detectable cases**
IL-12p70 (pg/ml) (>1.5)	38 (26.8)	4.35 (0.25‒390.77)
IFN-γ (pg/ml) (>1.6)	38 (26.8)	20.565 (0.84‒2237.85)
IL-1β (pg/ml) (>4.2)	25 (17.6)	24.49 (2.01‒1023.16)
IL-2 (pg/ml) (>16.4)	76 (53.5)	40.495 (8.35‒10781.05)
IL-4 (pg/ml) (>20.8)	74 (52.1)	31.82 (2.74‒1276.77)
IL-5 (pg/ml) (>1.6)	17 (12.0)	10.97 (0.29‒17042.32)
IL-6 (pg/ml) (>1.2)	41 (28.9)	8.41 (0.01‒163.63)
IL-9 (pg/ml) (>1.5)	26 (18.3)	6.02 (0.26‒1101.07)
IL-10 (pg/ml) (>1.9)	41 (28.9)	4.67 (0.07‒4669.57)
IL-13 (pg/ml) (>4.5)	90 (63.4)	38.425 (7.73‒539.93)
IL-17A (pg/ml) (>2.5)	42 (29.6)	26.035 (0.57‒1230.49)
IL-22 (pg/ml) (>43.3)	74 (52.1)	299.675 (5.99‒1963.32)
TNF-α (pg/ml) (>3.2)	45 (31.7)	13.94 (0.05‒1662.77)

^†^One patient had both HBV and HCV infection as causes of HCC.

Abbreviations: HCC, hepatocellular carcinoma; TACE, transarterial chemoembolization; HBV, hepatitis B virus; HCV, hepatitis C virus; AFP, alpha-fetoprotein; PIVKA-II, protein induced by vitamin K absence/antagonist-II PVT, portal vein thrombosis.

### Correlation of T cell cytokines or Tregs with clinical features

**Figure 1:**
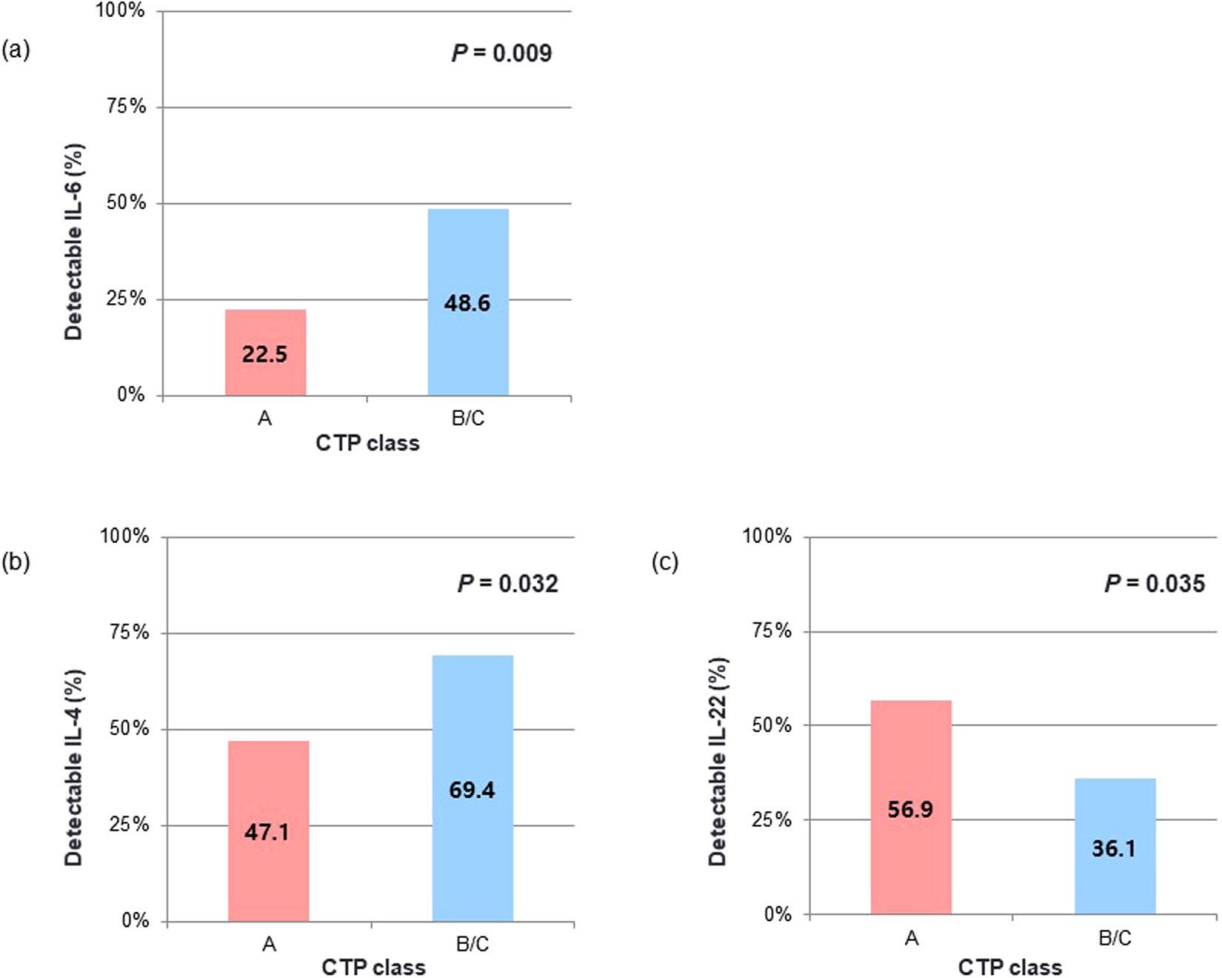
Comparison of the proportion of detectable T cell cytokine levels between patient group with CTP class A and that with B or C (**a**–**c**). CTP, Child-Turcotte-Pugh.

**Figure 2:**
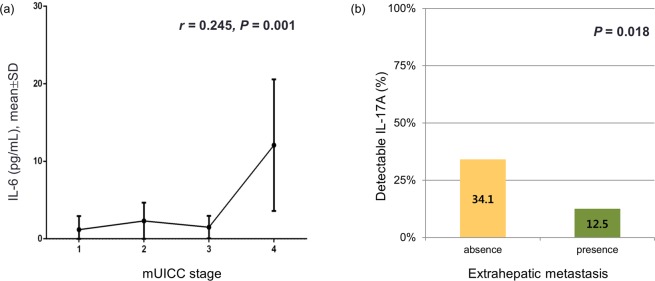
Difference in cytokine levels according to tumor stages. (**a**) There was significant correlation between serum IL-6 level and mUICC stages (*r* = 0.245, *P* = 0.001). (**b**) Proportion of detectable IL-17A was smaller in patients with extrahepatic metastasis than in patients without extrahepatic metastasis (P = 0.018). HCC, hepatocellular carcinoma.

Associations between levels of T cell cytokines or Tregs and laboratory parameters representing liver function were investigated. Patients with detectable IL-6 had significantly lower albumin (detectable IL-6, 3.3 ± 0.5 vs. undetectable IL-6, 3.7 ± 0.6, *P* < 0.001), higher bilirubin (1.6 ± 1.5 vs. 1.2 ± 0.8, *P* = 0.006), and higher Model for End-stage Liver Disease (MELD) scores (11 ± 3 vs. 9 ± 2, *P* < 0.001) than patients with undetectable IL-6. Patients with detectable IL-4 had lower albumin (detectable IL-4, 3.5 ± 0.6 vs. undetectable IL-4, 3.7 ± 0.6, *P* = 0.010) and higher ALT levels (58.0 ± 63.3 vs. 39.2 ± 23.8, *P* = 0.010) than those with undetectable IL-4. There were significant associations between Child-Turcotte-Pugh (CTP) class and IL-6, IL-4, and IL-22 (Fig. 1a–c). A significantly smaller proportion of patients with CTP class A had detectable IL-6 (*P* = 0.009) and IL-4 (*P* = 0.032) compared to patients with CTP class B or C. A larger proportion of patients with CTP class A had detectable IL-22 compared to patients with CTP class B or C (*P* = 0.035). Regarding tumor characteristics, detectable IL-6 was significantly associated with large tumor size (>5 cm, *P* = 0.001), multiple rather than solitary tumors (*P* = 0.008), and the presence of PVT (*P* < 0.001). There was significant correlation between IL-6 level and modified Union for International Cancer Control (mUICC) stage (r = 0.245, *P* = 0.001) (Fig. 2a). A smaller proportion of patients with extrahepatic metastasis had detectable IL-17A compared to patients without extrahepatic metastasis (*P* = 0.018) (Fig. 2b).

### Baseline cytokine levels as predictors for overall survival

**Figure 3:**
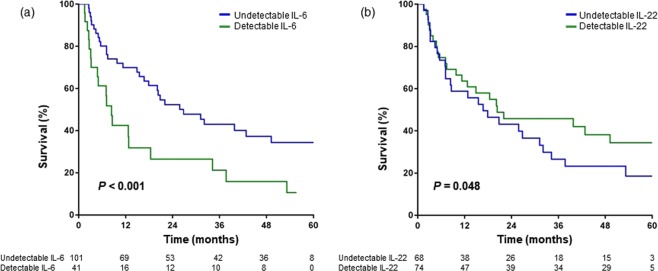
Kaplan-Meier curves of OS for patients with detectable and undetectable cytokine levels. (**a**) Survival according to detectability of IL-6. (**b**) Survival according to detectability of IL-22. OS, overall survival.

During a mean follow-up period of 28.1 ± 22.4 months, 79 (55.6%) of 142 patients died, 38 (26.8%) remained alive, and 25 (17.6%) were lost to follow-up. The cumulative survival rates were 55.9%, 47.1%, and 36.7% at 2, 3, and 5 years, respectively. The potential effects of baseline serum levels of CD4+ T cell cytokines and Tregs on overall survival (OS) were evaluated. The cumulative OS rate of patients with detectable IL-6 was significantly lower than that of patients with undetectable IL-6 (*P* < 0.001) (Fig. 3a). Conversely, patients with detectable IL-22 had significantly higher survival rate than those with undetectable IL-22 (*P* = 0.048) (Fig. 3b). In univariate analysis, CTP class B/C (*P* < 0.001), alpha-fetoprotein (AFP) level (*P* < 0.001), prothrombin induced by vitamin K absence-II (PIVKA-II) (*P* < 0.001), mUICC stage IV (*P* < 0.001), detectable IL-6 (*P* < 0.001) and IL-22 (*P* = 0.050) were predictors for poor OS. Multivariate analysis identified CTP class B/C (Hazard ratio [HR], 2.52; 95% confidence interval [CI], 1.48‒4.31; *P* < 0.001), PIVKA-II (HR, 1.94; 95% CI, 1.09‒3.46; *P* = 0.025), mUICC stage IV (HR, 7.61; 95% CI, 4.03‒14.37; *P* < 0.001) and detectable IL-6 (Hazard ratio [HR], 1.68; 95% confidence interval [CI], 1.00‒2.82; *P* = 0.048) as independently predictive of poor OS (Table 2).

**Table 2:** Prognostic factors of overall survival.

Baseline characteristics	Univariate	Multivariate		
	*P*	HR	95% CI	*P*
Sex, male	0.065			
Age > 60 years	0.778			
Etiology, viral vs. non-viral	0.150			
CTP class B/C vs. A	<0.001	2.52	1.48‒4.31	0.001
AFP > 60 (ng/mL)	<0.001			0.717
PIVKA-II > 185 (mAU/mL)	<0.001	1.94	1.09‒3.46	0.025
mUICC stage IV	<0.001	7.61	4.03‒14.37	<0.001
Detectability of cytokine				
IL-12p70	0.375			
IFN-γ	0.464			
IL-1β	0.449			
IL-2	0.285			
IL-4	0.692			
IL-5	0.066			
IL-6	<0.001	1.68	1.00‒2.82	0.048
IL-9	0.388			
IL-10	0.969			
IL-13	0.931			
IL-17A	0.517			
IL-22	0.050			0.728
TNF-α	0.073			

Abbreviations: OS, overall survival; CI, confidence interval; HR, hazard ratio; AFP, alpha-fetoprotein; PIVKA-II, prothrombin induced by vitamin K absence-II; mUICC, modified Union for International Cancer Control.

### Effect of changes in CD4+ T cell subsets on OS following TACE

**Figure 4:**
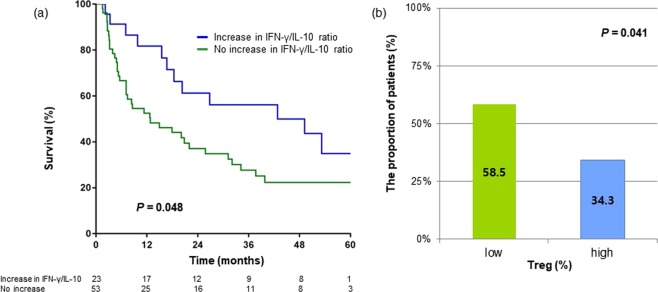
(**a**) Overall survival in patients who had increased IFN-γ/IL-10 ratio following TACE versus those who did not. (**b**) Proportion of patients with Th1/Th2 ratios that increased by more than one third following TACE. More patients with low Tregs at baseline had increased Th1/Th2 ratios after TACE (P = 0.041). Th1, type 1 helper T cell; Th2, type 2 helper T cell; TACE, transarterial chemoembolization; Tregs, regulatory T cells.

Follow-up measurements of multiple T cell cytokines were available in 76 patients. Dynamic changes in CD4+ T cell subsets following first TACE were evaluated. Changes in Th1/Th2 cytokine ratios (IL-1/IL-4, IL-2/IL-4, IFN-γ/IL-4, IL-12/IL-4, IL-1/IL-5, IL-2/IL-5, IFN-γ/IL-5, IL-12/IL-5, IL-1/IL-10, IL-2/IL-10, IFN-γ/IL-10, and IL-12/IL-10 ratio) and IL-6 level were divided into groups of increase in value and no increase in value. Patients who had increased IFN-γ/IL-10 ratio had significantly better survival than those who did not (*P* = 0.048) (Fig. 4a). In multivariate analysis, increased IFN-γ/IL-10 ratio (OR, 0.45; 95% CI, 0.23−0.88; *P* = 0.020) and IL-6 level (OR, 1.95; 95% CI, 1.05−3.64; *P* = 0.035) were identified as independently predictive factors for poor OS (Table 3).

**Table 3:** Association between changes in Th1/Th2 cytokine ratio and IL-6 level and patient survival.

Increase in Th1/Th2 ratio or IL-6	Univariate	Multivariate		
level after TACE	*P*	HR	95% CI	*P*
IL-1/IL-4	0.110			
IL-2/IL-4	0.863			
IFN-γ/IL-4	0.982			
IL-12/IL-4	0.522			
IL-1/IL-5	0.881			
IL-2/IL-5	0.652			
IFN-γ/IL-5	0.823			
IL-12/IL-5	0.947			
IL-1/IL-10	0.057			0.298
IL-2/IL-10	0.551			
IFN-γ/IL-10	0.041	0.45	0.23–0.88	0.020
IL-12/IL-10	0.104			
IL-6	0.115	1.95	1.05–3.64	0.035

Abbreviations: Th1, type 1 helper T cell; Th2, type 2 helper T cell; TACE, transarterial chemoembolization; CI, confidence interval; HR, hazard ratio.

We then evaluated whether baseline Tregs influence changes in Th1/Th2 balance following TACE. Patients were divided into two groups based on median Treg levels at baseline, and the proportions of patients who showed an increase in at least 5 out of measured Th1/Th2 ratios were compared between the two groups. As a result, 24 (58.5%) of the 41 patients in the low Tregs group had an increase in Th1/Th2 ratio following first TACE, compared to 12 (34.3%) of the 35 patients in the high Tregs group (*P* = 0.041) (Fig. 4). This suggests that baseline Tregs level could affect the direction in which Th1/Th2 balance changes and is related to the prognosis of HCC patients.

## Discussion

Although knowledge has accumulated regarding the role of immune cells and related cytokines in HCC, there is limited clinical use because each immune regulator is usually studied individually or from tumor tissues that are not easily accessible. We comprehensively analyzed the prognostic roles of multiple T cell cytokines and Tregs in patients with HCC treated by TACE using whole blood samples. IL-4 and IL-22 were related to hepatic reserve, while IL-17A was associated with extrahepatic metastasis. IL-6 was the most significant cytokine in this study related to liver function, tumor characteristics, and OS of HCC patients. Furthermore, increased IL-6 level after TACE significantly predicted poor OS. When Th1/Th2 balance shifted to Th1 dominance after TACE, outcomes were favorable. Baseline Tregs levels could affect the balance of Th1/Th2 cytokines.

IL-6 is a pro-inflammatory cytokine with deleterious effects in several malignancies and inflammatory disorders^12,13^. In a previous study of patients with HCC, serum IL-6 level was significantly associated with advanced tumor characteristics, including large tumor size, presence of PVT, and extrahepatic metastasis, and IL-6 has also been proven as a significant predictor for unfavorable outcome^14^. IL-6 has been suggested as a marker for HCC with higher efficacy than AFP^15^. This study also showed a close relationship between detectable IL-6 and tumor burden, and the mean serum level of IL-6 in patients with mUICC stage IV was significantly higher than that of patients with mUICC stage I-III. These features contribute to the function of IL-6 as an independent predictor of poor OS. Additionally, CTP or MELD score was higher in patients with detectable IL-6, indicating that IL-6 level is correlated with liver function. Senescent hepatocytes, which account for as many as 80% of hepatocytes in advanced chronic liver disease, might contribute to increased IL-6 expression^16,17^.

In our analysis, cumulative OS was significantly higher in patients with detectable IL-22 than in patients with undetectable IL-22. The liver is an important target of IL-22, which generally demonstrates hepatoprotective properties through anti-fibrotic and regenerative effects during liver infection and damage^18^. However, IL-22 was previously demonstrated as a tumor promoting or negative prognostic factor in patients with HCC. A previous study showed that patients with HCC and high serum IL-22 concentration had poorer prognosis than patients with a low level of IL-22^19^. Sustained IL-22 elevation and subsequent STAT3 activation were found with up-regulation of downstream genes in HCC mouse models, promoting tumor development and metastasis^20^. IL-22 is secreted from Th22, Th17, and Th1 cells^21^, which can all act differently in HCC development. The role of IL-22 reportedly varied depending on the type or degree of liver disease^22,23^. Further study is needed to elucidate the function of IL-22 in hepatocarcinogenesis.

Although no single cytokine from Th1 or Th2 cells had prognostic value for OS in this study, we showed that dynamic changes of Th1/Th2 balance after TACE were associated with OS. An increase in IFN-γ/IL-10 ratio after TACE was independently predictive of prolonged OS, and increased IL-1/IL-10 ratio also tended to predict prolonged OS. These findings were consistent with previous studies in which Th2 response had a dominant role in tumor development and progression. Thus, tumor microenvironment shifted to Th1 dominance could have beneficial effects on tumor regression^24,25^. In patients with advanced HCC treated with intra-arterial chemotherapy, the proportion of Th2 cells was significantly higher both before and after treatment in patients with progressive tumor response than in patients with partial response or stable disease^26^.

In this study, a larger proportion of patients with low Tregs level at baseline changed to Th1 dominance after TACE compared to patients with a high baseline Tregs level. Tregs are functionally immunosuppressive and could directly or indirectly inhibit the proliferation of other CD4+ T cells via cytokines to further decrease local immunologic responses to tumor cells^27^. Similarly, a previous study of patients with colorectal cancer showed that Tregs suppressed effector CD4+ T cell responses to tumor-associated antigen before tumor resection, and the suppression was associated with tumor recurrence^28^.

This study has some limitations. Although a multiplex bead analysis used in our analysis allowed simultaneous measurement of T cell cytokines, the number of undetectable cytokines was relatively high with a high cut-off value, and Tregs levels (%) were relatively low compared to other studies. This might have lowered the sensitivity and prognostic power for T cell cytokines and Tregs to correlate with the clinical characteristics and prognosis of HCC. Changes in T cell cytokines following first TACE were insufficiently evaluated because subsequent measurements of cytokines were available in only a small number of patients. Nevertheless, this comprehensive analysis shows that T cell cytokines and Tregs have distinctive properties that correlate well with prognosis of patients with HCC.

In conclusion, T cell cytokines play an important role in hepatic function, tumor characteristics, and prognosis in patients with HCC. IL-6 was among the most significant predictive factors for poor OS in patients undergoing TACE. Baseline Tregs level could influence the direction of Th1 or Th2 response following treatment. A shift toward increased Th1 response and no increase in IL-6 level could exert favorable immunologic effects on HCC prognosis. This study showed the potential role of multiple cytokines as biomarkers for HCC, and the demand for multiple cytokine biomarkers will increase in the upcoming era of immunotherapy.

## Methods

### Patients

This study evaluated patients newly diagnosed with unresectable HCC who underwent TACE as the 1st treatment between March 2011 and December 2012 at a tertiary center in South Korea. In our center, TACE was mainly selected for patients with CTP class A or B who had unresectable HCC without main PVT. Additionally, patients who had small tumors but ineligible for radiofrequency ablation or percutaneous ethanol injection therapy were also considered to receive TACE. Some patients with PVT or extrahepatic metastasis were evaluated by a multidisciplinary team for TACE or TACE combined with radiotherapy if clinically warranted. Patients treated with other modalities including systemic therapy were excluded from the analysis. HCC was diagnosed by histologic evidence or typical radiologic findings and/or elevated AFP in patients with risk factors for HCC. Staging of HCC was complementarily determined based on mUICC and Barcelona Clinic Liver Cancer staging according to guidelines of The Korean Liver Cancer Association and National Cancer Center^29^. Patients were followed up until death or February 2017. Each patient provided informed consent to participate in the study. This study was approved by the Ethics Committees of The Catholic University of Korea in accordance with the 1975 Declaration of Helsinki.

### Treatment and laboratory data

The intra-arterial chemotherapeutic agents used were doxorubicin (50 mg) or combined epirubicin (50 mg) and cisplatin (60 mg) in a mixture of lipiodol (5–10 mL). TACE sessions were repeated every 1–2 months until complete response by one experienced interventional radiologist. Clinical and laboratory data of enrolled patients were obtained at the time of diagnosis of HCC and routinely measured during the treatment period including gender, age, hematologic and biochemical parameters and tumor status.

### Assays

Whole blood for analyzing CD4+ T cell cytokines was obtained twice from enrolled patients, at the time of diagnosis and 1‒2 months after first TACE, and was stored at −20 °C. Serum concentrations of CD4+ T cell cytokines (IL-1β, IL-2, IL-4, IL-5, IL-6, IL-9, IL-10, IL-12p70, IL-13, IL-17A, IL-22, IFN-γ, and TNF-α) were measured by enzyme linked immunosorbent assay (Flowcytomix™ Multiplex) using Multiplex cytometric bead immunoassay (eBioscience, San Diego, CA, USA). Whole blood obtained at baseline was also used to measure Tregs using a Human Regulatory T-cell Staining Kit (eBioscience, San Diego). Peripheral blood mononuclear cells were isolated by centrifugation with Ficoll-Hypaque gradients and incubated with anti-CD4-FITC and anti-CD25-APC monoclonal antibodies for 30 minutes at 4 °C.

### Statistical analyses

All data were expressed as the mean ± SD or median (range). The chi-square test or Fisher’s exact test was used to compare categorical variables. The cumulative rate of overall survival was estimated using the Kaplan-Meier method, and differences were evaluated with the log-rank test. Continuous variables were transformed into two-level categorical data, based on their median values. Univariate and multivariate analyses were performed using Cox proportional hazard model to identify prognostic factors for OS. A two-sided P value < 0.05 was considered statistically significant. Data were analyzed using SPSS 20.0 (SPSS Inc., Chicago, IL, USA).

## Data Availability

The datasets generated during and/or analysed during the current study are available from the corresponding author on reasonable request. All data generated or analysed during this study are included in this published article.
